# Genetic Variants of *APOC3* Promoter and *HLA-B* Genes in an HIV Infected Cohort in Northern South Africa: A Pilot Study

**DOI:** 10.3390/ijms150711403

**Published:** 2014-06-26

**Authors:** Tracy Masebe, Pascal Obong Bessong, Roland Ndip Ndip, Debra Meyer

**Affiliations:** 1 HIV/AIDS & Global Health Research Programme, Department of Microbiology, University of Venda, Thohoyandou 0950, South Africa; E-Mail: tracy.masebe@univen.ac.za; 2 Department of Microbiology and Parasitology, Faculty of Science, University of Buea, Buea Box 63, Cameroon; E-Mail: ndip3@yahoo.com; 3 Department of Biochemistry and Microbiology, Faculty of Science and Agriculture, University of Fort Hare, Alice 5700, South Africa; 4 Department of Biochemistry, University of Pretoria, Pretoria 0002, South Africa; E-Mail: debra.meyer@up.ac.za

**Keywords:** genetic variants, APOC3, HLA-B, highly active antiretroviral therapy, lipid disorders, South Africa

## Abstract

Metabolic disorders and hypersensitivities affect tolerability and impact adherence to highly active antiretroviral therapy (HAART). The aim of this study was to determine the prevalence of C-482T/T-455C variants in the Apolipoprotein C3 (APOC3) promoter gene and Human leukocyte antigen (HLA)-B*57:01, known to impact lipid metabolic disorders and hypersensitivity respectively; and to correlate genotypes with gender, CD4^+^ cell count and viral load in an HIV infected cohort in northern South Africa. Frequencies of *C*-482 and *T*-455 polymorphisms in APOC3 were determined by restriction fragment length polymorphism analysis. Allele determination for HLA-B was performed with Assign SBT software in an HLA library. Analysis of APOC3 *C*-482 site revealed a prevalence of 196/199 (98.5%) for CC, 1/199 (0.5%) for CT and 2/199 (1.0%) for TT genotype (*p* = 0.000 with 1° of freedom; χ^2^ = 126.551). For the T-455 site, prevalences were: 69/199 (35%) for TT and 130/199 (65%) for the CC genotype (*p* = 0.000 with 1° of freedom; χ^2^ = 199). There was no association between gender and the presence of −482 (*p* = 1; χ^2^ = 0.00001) or −455 genotypes (*p* = 0.1628; χ^2^ = 1.9842). There was no significant difference in the increase in CD4^+^ cell count irrespective of genotypes. Significant increases in CD4^+^ cell count were observed in males and females considering the −455C genotype, but not in males for the −455T genotype. Viral load decreases were significant with the −455C and −482C genotypes irrespective of gender. HLA-B*57:01 was not identified in the study cohort. The apparently high prevalence of APOC3 *T*-455CC genotype needs confirmation with a larger samples size and triglyceride measurements to support screening of patients to pre-empt HAART associated lipid disorders.

## 1. Introduction

The clinical benefit of highly active antiretroviral therapy (HAART) is tempered with adverse drug events that have emerged as a major concern for long-term successful management of HIV infection [1]. Currently, about 83% of patients on HAART, experience metabolic disorders, as compared to approximately 40%–50% of cases prior to HAART [2,3].

Development of metabolic disorders and hypersensitivity reactions affects HAART tolerability and adherence, and may lead to dose interruptions, therapy discontinuation, and/or decrease in life expectancy [4]. Although HAART is implicated in the development of such disorders, there is considerable inter-individual variability in patient outcomes in terms of drug disposition, drug efficacy and adverse events. In addition, factors such as host genetics and ethnicity are thought to play a significant role [1,5,6,7]. Of note is the human leukocyte antigen subtype B*57:01; a strong predictor of abacavir hypersensitivity reactions [6,7], as well as variants of the Apolipoprotein C3 (*APOC3*) promoter region such as *C*-482T and *T*-455C associated with development of hyperlipidemia [8,9]. 

*Apolipoprotein C3* gene is involved in transport and clearance of chylomicron remnants, and very low and high density lipoproteins from the bloodstream. The human *APOC3* is mapped to chromosome 11q23, closely linked to APOA1 and APOA4 genes [10]. The presence of polymorphisms at the insulin responsive element on the promoter region leads to over-expression of the protein [11]. The *APOC3* −482 and −455 promoter polymorphisms have reduced affinity for nuclear transcription factors mediating insulin responses associated with insulin resistance. A change from thymine to cysteine at position −455 is associated with increased triglyceride levels, a risk factor for cardiovascular disease. The cysteine to thymine change at position −482 is associated with dyslipidemia and insulin resistance [12].

Therapy-associated lipid disorders are common in HIV/AIDS patients and have been linked to polymorphisms on apolipoproteins [13]. Dyslipidemia and lipodystrophy occur in some HIV-infected patients despite similar drug exposures and comparable demographic, immunologic and virologic characteristics. Host genetic factors may account for this variability in responses to drugs [14]. However, non nucleoside reverse transcriptase inhibitors (NRTIs), such as stavudine and didanosine, exhibit a number of safety concerns that limit their clinical use and these drugs are associated with the development of peripheral neuropathy, dyslipidemia and lactic acidosis [15].

Pathogenesis of a number of drug hypersensitivity reactions involves restricted presentation of drugs to major histocompatibility complex (MHC) molecules prior to T cell presentation [16]. The consequence of this is the development of hypersensitive reactions to a given drug. Alleles of MHC and their frequencies in different racial groups are implicated in the development of hypersensitive reactions in patients on abacavir and nevirapine within the first six weeks of treatment [17,18,19]. Although low frequencies of HLA-B*57:01 have been observed in other African populations, data on the prevalence of this allele in South African populations, particularly in the northern region with a different ethnicity is scanty. Furthermore, there are several reports on the associations of variants of the *APOC3* promoter region with the development of metabolic disorders [20], and the link of HLA-B*5701 allele with Abacavir hypersensitivity but little is known about the genetics of the population in northern South Africa in this regard. Thus, the aim of this study was to determine the presence of single nucleotide polymorphisms (SNP) on promoter regions of the *APOC3* gene; and the prevalence of HLA-B*57:01.

## 2. Results

### 2.1. Study Population and Clinical Data

The study population (*n* = 206) comprised 146 females. The mean age was 36.8 years (range 18–59). The treated participants were on stavudine-based therapy, with 60 patients on regimen 1a (stavudine, lamivudine and efavirenz) and 34 on regimen 1b (stavudine, lamivudine and nevirapine). One hundred and twelve patients were not on therapy at the time of the study. Clinical information such as CD4^+^ cell count and viral load measurements were available for patients who were followed up for 12 months after therapy initiation. The CD4^+^ cell counts and viral load values prior to therapy were respectively 1–700 cells/mm^3^ and 1727–1,797,648 copies/mL. Statistical analysis was done for 53 patients (of the 94 who were on treatment) who had complete immunological and virologic parameters at 6 months post therapy. They all experienced better immune recovery. Forty one of the patients either discontinued therapy due to development of side effects, relocated (*n* = 1) or died (*n* = 11) over a 12 month follow up period. Available clinical records of patients were consulted and the following most commonly ARV-induced adverse reactions were noted: lymphadenopathy, peripheral neuropathy, hyperlactaemia, dryness of skin and skin rash.

Four of 94 patients experienced adverse drug events. Three of them developed hyperlactemia and this resulted in a treatment switch from regimen 1a to regimen 2a (Zidovudine, didanosine and Lopinavir/ritonavir). Two of these patients died, one from heart failure. Lack of adherence was suspected in one patient who experienced dryness of skin and treatment was switched from regimen 1a to tenofovir-lamivudine-lopinavir/ritonavir. However, there was no improvement and a further switch to tenofovir-lamivudine-didanosine was made.

### 2.2. APOC3 Promoter Region Amplification and RFLP Results

**Figure 1:**
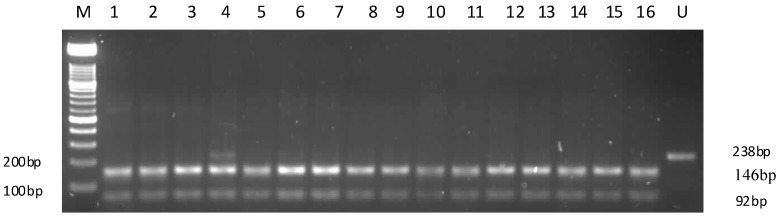
A representative 2% agarose gel of restriction digestion products with MspI for −482 of *APOC3* promoter region genotyping. The expected size of amplified product was 238 bp. Restriction patterns obtained with MspI were as follows: Lane M: 100 bp molecular marker; Lanes 1–3 and 5–16, wild type −482 CC genotype; Lane 4, CT, heterozygous genotype; and Lane U: undigested sample (no enzyme added). The presence of a restriction site is indicated by two or three bands.

**Figure 2:**
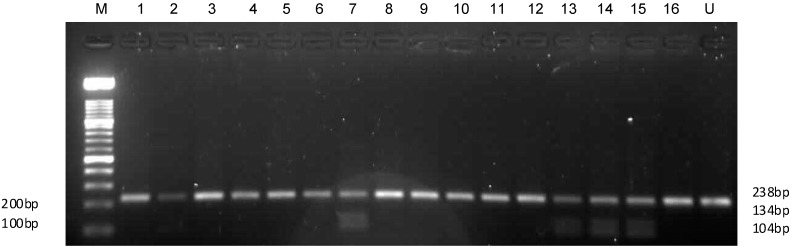
A representative 2% agarose gel of restriction digestion products with FokI for *APOC3* −455 genotyping. Restriction patterns obtained with FokI were as follows: Lane M, 100 bp molecular marker; Lanes: 1–6, 8–12 and 16: CC variant genotype (no restriction site); Lanes 7 and 13–15, wild type TT genotype; and Lane U: undigested sample (no enzyme added). One band indicates the absence of a restriction site; three bands indicate the presence of a restriction site.

Amplified products were obtained for all the subjects, but RFLP results were available for 199 samples (*n* = 206). Genotypes of APOC3 promoter gene (238 pb) were assigned based on restriction patterns and were classified as follows: For *C*-482 site (MspI): CC (homozygous, wild), CT (heterozygous), TT (homozygous). For *T*-455 site (FokI), TT (homozygous, wild), TC (heterozygous), CC (homozygous) (Figure 1 and Figure 2).

### 2.3. Sequencing

Sequencing of a subset (*n* = 12) of amplified products of the *APOC3* promoter region was carried out to corroborate the RFLP results. Sequences were aligned with wild type (178839|gb|M60674.1) and variant (Hagrid 0102) *APOC3* promoter sequences obtained from GenBank. A total of 66.6% of the sequences were classified as variant T allele, 16.7% as wild type C allele and 16.7% as heterozygotes of wild and variant alleles at the −482 MspI site. At the −455 FokI site, 58.3% of the sequences were classified as variant C allele, 8.33% as heterozygote of wild and variant allele, and 33.33% as wild type T allele.

### 2.4. Genotype Frequencies

Allele and genotype relative frequencies were estimated through genotype counting. A 98.7% and a 1.3% prevalence of C wild type allele and T variant allele were observed at the −482 polymorphic site respectively. The genotype frequencies were as follows; 98.5% CC genotype, 0.5% CT genotype and 1% TT genotype. For the −455 restriction site, prevalence rates of 35% for the wild type T allele and 65% for the variant type C allele were observed. The genotype frequencies were 35% and 65% for TT and CC respectively. Genotypes identified at the APOC3 gene locus at positions C-482 and T-455 resulted in the presence of the following haplotypes: −482CC/−455TT (69/199), −482CC/−455CC (127/199), −482CC/−455CC (1/199) and −482TT/−455CC (2/199).

### 2.5. Statistical Analyses

There was no significant difference in the increase in CD4^+^ cell count and decrease in plasma viral load among patients irrespective of APOC3 −482 (CC, CT and TT) and APOC3 −455 (TT, TC and CC) genotypes. Using the Tukey’s Studentized Range Test at the 0.05 confidence level, significant increases in CD4^+^ cell count were observed over 6 months in males and females for the *APOC3* −455C genotype, while significant increases in CD4^+^ cell count was observed among females and not in males for the *APOC3* −455T genotype. Viral load decreases were significant for both males and females when the *APOC3* −455C genotype was considered. However, there was a significant range decrease in males and not in females with the *APOC3* −455T genotype. For the −482C genotypes there were significant range increases in CD4^+^ cell counts and range decreases in viral load irrespective of gender at the 0.05 confidence level. Equally, there was no statistical significant relationship between genders and the presence of −482 genotypes (*p* = 1; χ^2^ = 0.00001) or −455 genotypes (*p* = 0.1628; χ^2^ = 1.9842).

Mauchly’s test for phericity assumption suggested that there was sufficient evidence to indicate significant viral load decreases over time, but insignificant CD4^+^ cell count increases. On the other hand, repeated measures of analysis suggest that there were significant CD4^+^ cell count increases and viral load decreases over time for *APOC3* genotypes −482CC, −455TT and −455CC (χ^2^ and *p* values are presented in Table 1.

Observed allele and genotype frequencies did not deviate from those expected under the Hardy-Weinberg equilibrium (HWE) at the −482 polymorphic site (χ^2^ = 126.5511; *p* = 0.0000 with 1° of freedom). However, at the −455 polymorphic site, the observed genotypes deviated from those predicted by the Hardy-Weinberg equation (χ^2^ =199; *p* = 0.0000 with 1° of freedom). The distribution of alleles and genotype frequencies is presented in Table 2.

### 2.6. HLA Amplification and Typing Results

DNA was successfully amplified from 183 subjects (88.8%). Upon sequencing, 113 (63%) sequences which contained both exons 2 and 3 were used for HLA typing. Upon HLA typing using Assign SBT software, 16 alleles were detected from 40 different individuals, and no allele assignment was possible for 73 samples which contained several nucleotide mismatches when compared to known HLA-B reference sequences obtained from an HLA library compiled from the 2.17.1.0 release of the IMGT/HLA Database.

**Table 1:** Repeated measures of analysis of *APOC3* genotypes of study participants with CD4 and viral load parameters. A *p* value of <0.05 was considered significant. Values are only for 53 patients for whom viral load and CD4^+^ cell count measurements were available. No analysis was done for *APOC3* −482CT and TT genotypes as very few individuals were identified with these genotypes. χ^2^ value indicates the difference between expected and observed genotype frequencies

*APOC3* Genotypes	Mauchly’s Test for Sphericity		Repeated Measures		Mauchly’s Test for Sphericity		Repeated Measures	
	CD4		CD4		VLD		VLD	
	χ^2^	*p* Value	*F* Value	*p* Value	χ^2^	*p* Value	*p* Value	*F* Value
−482CC	4.1177	0.1276	56.15	<0.0001	217.366	0.0001	<0.0001	21.48
−482CT	NA	NA	NA	NA	NA	NA	NA	NA
−482TT	NA	NA	NA	NA	NA	NA	NA	NA
−455TT	3.1254	0.2096	32.25	<0.0001	92.1284	<0.0001	0.0119	8.03
−455CC	4.0513	0.1319	32.68	<0.0001	99.7070	<0.0001	<0.0001	25.02

**Table 2:** Observed and expected allele and genotype frequencies of *APOC3* promoter region in South African subjects determined by the Hardy-Weinberg method. Hardy-Weinberg Equilibrium is not applicable if there are less than five events in the genotypic analysis. χ^2^ value indicates the difference between expected and observed genotype frequencies; Where *p* < 0.05 the expected and observed data are not consisted with HWE.

Genotypes	Observed Genotypes	Expected Genotypes	Genotype Frequency	χ^2^	*p* Value	Alleles	Expected Alleles	Allele Frequency
*APOC3* −482CC	196	194.0	0.985	126.5511103	0.00	*APOC3* −482C	0.99	0.9875
*APOC3* −482CT	01	4.9	0.005			*APOC3* −482T	0.01	0.0125
*APOC3* −482TT	02	0.0	0.010			*APOC3* −455T	0.65	0.65
*APOC3* −455TT	69	23.9	0.35	199	0.00	*APOC3* −455C	0.35	0.35
*APOC3* −455TC	0	90.2	0					
*APOC3* −455CC	130	84.9	0.65					

The detected alleles were as follows: HLA-B*07:02:01 (2/40), HLA-B*15:01:01 (1/40), HLA-B*15:03:01 (14/40), HLA-B*15:17:01:01 (1/40), HLA-B*15:10:01 (1/40), HLA-B*15:18:01 (1/40), HLA-B*18:01:01 (2/40), HLA-B*41:01 (1/40), HLA-B*44:03:01 (1/40), HLA-B*44:03:02 (1/40), HLA-B*45:01 (3/40), HLA-B*45:04 (1/40), HLA-B*53:01:01 (2/40), HLA-B*58:01:01 (6/40), HLA-B*58:02 (2/40) and HLA-B*81:01 (1/40).

## 3. Discussion

Host genetic factors associated with drug toxicities represent major obstacles to optimal treatment management in AIDS patients. Genetic biomarkers on the *APOC3* (*C*-482T and *T*-455C) gene have been associated with hypertriglyceridemia in several distinct populations and their presence reduces *APOC3* protein expression [20]. On the other hand, the HLA-B*57:01 allele has been associated with abacavir hypersensitivity. Development of lipid disorders and drug-associated hypersensitivity reactions may lead to therapy withdrawal. The aim of the current investigation was to determine the presence and frequency of *C*-482T and *T*-455C in the *APOC3* promoter region, and the frequency of the HLA-B*57:01 allele in a cohort of black South African HIV-1 patients in an attempt to identify patients at risk of developing untoward metabolic effects such as hyperlipidemia and abacavir associated hypersensitivity.

The study participants were on a stavudine-based first line therapy. About 2% of the study participants had at least one side effect (hyperlactaemia, heart failure, or dry skin) linked to the use of stavudine, although 35% of the patients harboured the *APOC3* −455CC allele. In like manner, although more than 60% of the subjects were identified with −482CC/−455CC haplotype only 4 patients showed associated side effects such as hyperlactemia and peripheral neuropathy, and treatment was subsequently switched. A high prevalence of *C*-482 allele and a low prevalence of the *APOC3* *T*-455 allele were observed. Wild type alleles at these two polymorphic sites are associated with low risk for metabolic complications, and Africans have been shown to have better lipid profiles compared to other races [9]. 

The observed prevalence of wild type allele −482 in the current study is comparable to observations in other studies where higher prevalence of wild type alleles and their association with better lipid profiles were reported in individuals of African descent [21,22,23]. Foulkes and colleagues [20] in their investigations also acknowledged race/ethnicity as a highly significant predictor of plasma lipids in HIV patients. They observed significantly lower triglyceride and increased levels of high density lipoprotein cholesterol in blacks/non Hispanics compared to Caucasians and Hispanics. The lower rates of *APOC3* −482TT and −455CC wild type alleles are identified as predominant factors underlying the apparent genetic resistance to metabolic diseases [24]. In the current study, a significantly lower frequency of *APOC3* −482T variant was observed compared to *APOC3* −455C. Variant alleles (−482T and −455C) of the *APOC3* promoter region are associated with higher triglyceride levels [23], as such individuals with these alleles may be at higher risk of developing metabolic disorders [25]. It has been inferred that both −482T and −455C alleles are unresponsive to insulin; hence, inappropriately increased levels of APOC3 are observed in subjects with these alleles [22,25]. Petersen *et al.* [26] also observed an increase in plasma triglycerides in Asian Indian and non Asian Indian subjects (white, Asian, Hispanic and black) with *C*-482T and *T*-455C *APOC3* variant alleles. The −482TT/−455CC haplotype was also observed in present study, which has been shown elsewhere to attenuate inhibitor action of insulin on APOC3 gene expression, with subsequent increases in plasma lipid levels [27]. In AIDS patients, the presence of a −455CC genotype is associated with a more severe dyslipidemia and lower risk of lipoatrophy development [28].

Herein, analysis of immunologic and virologic parameters showed that patients responded well to therapy and there was no statistically significant difference in immunologic (*p* < 0.001) and virologic (*p* < 0.001) recovery of patients when associated with a specific genotype of either *APOC3* −482 or −455. A lack of association has also been observed in studies involving patients with or without lipodystrophy [13]. Variant alleles of *APOC3* contribute to the development of dyslipidemia in AIDS patients even prior to HAART initiation. In the current observation, a low prevalence of −482T and a high prevalence of *APOC3* −455C variants were observed. Identification of such variants predisposing patients to lipid disorders is an indication that performing genotypic tests to identify biomarkers prior to therapy may be considered. 

In the study population reported here, all the HLA-B types observed have been identified in South African, Kenyan and Cameroonian populations, confirming their prevalence in African populations [28,29,30]. The prevalent allele HLA-B*15:03:01 is of African origin and has been observed in other African populations in frequencies ranging from 8% to 22%. Variations in African populations could reflect diverse genetic backgrounds of these groups [31,32].

Ambiguous typing results were obtained for the following HLA-B alleles: B*45:01, B*18:01:01, B*58:01:01, B*15:01:01, B*15:03:01, B*07:02:01 and B*81:01. Some of the sequences contained incomplete exons 2 and 3 and this might have led to ambiguous results. To resolve these ambiguities, sequencing of respective exons 1, 3 and 4 could have been necessary. However, if ambiguities are due to polymorphisms that do not alter protein function, it may not be necessary to re-sequence the recommended exon [33]. None of the study participants were identified with HLA-B*57:01 allele. However, related alleles, HLA-B*57:02:01 and HLA-B*58:01:01 alleles were identified in 0.25% and 15% of the current study participants respectively. These two alleles cannot stimulate specific CD8^+^ T cell response as compared to HLA-B*57:01, and thus their presence may not be associated with development of hypersensitive reactions [34,35].

A limitation of the current study is the small sample size and the investigation of specific SNP in *APOC3* and *HLA*. Genome wide association studies may be required to confirm these findings since additional polymorphisms may be implicated in lipid metabolic disorders and hypersensitivity to antiretrovirals [36]. These further studies will also need to look at variability in the different ethnic groups. 

## 4. Experimental Section

### 4.1. Study Population and DNA Extraction

The study population comprised a cohort of black South African patients recruited from two AIDS treatments sites in northern South Africa. The study protocol was approved by the University of Venda Research Ethics Committee (Study number SMNS/09/MBY/003). The experiments were undertaken with the understanding and written consent of each subject, and that the study conforms to the Code of Ethics of the World Medical Association (Declaration of Helsinki). Details on the study demographics, sample collection and genomic DNA extraction procedures have been described [37].

### 4.2. Amplification of the APOC3 Promoter Region

Genomic DNA was amplified by polymerase chain reaction (PCR) from 206 study participants. The following primers were used to amplify a 238 bp gene fragment: *APOC3* −482 5'-GGATTGAAACCCAGAGATGGAGGTG-3' (sense) and −455 5'-TTCACACTGGAATTTCAGGCC-3' (antisense) [11]. The PCR reaction mixture comprised a final concentration of 4 μM of each primer, 200 μM dNTPs, 4 U FastStart Taq polymerase (Roche Diagnostics, Mannheim, Germany), 5 µL 10× PCR buffer (MgCl_2_, 25 mM), 5 μL of DNA template and nuclease free water in a 50 μL total reaction volume. Cycling conditions comprised an initial denaturation at 94 °C for 3 min, followed by 35 cycles of denaturation at 94 °C for 2 min, annealing at 55 °C for 1 min, elongation at 72 °C for 2 min and a final elongation at 72 °C for 10 min. The resulting PCR products were resolved by electrophoresis on a 1% Ethidium bromide-stained agarose gel (Invitrogen, Carlsbad, CA, USA).

### 4.3. Genotyping of APOC3 Promoter Region

Amplified DNA was purified with a Qiagen DNA purification kit (Qiagen, Valencia, CA, USA) according to the manufacturer’s instructions. Genotypes of *APOC3* at −482C and −455T were determined by RFLP. Restriction enzymes, MspI (restriction site: 5'-C^CGG-3') and FokI (restriction site: 5'-GGATG(N)_9_^-3', Fermentas, Vilnius, Lithuania) were used for the *C*-482 and *T*-455 restriction sites respectively. Briefly, the RFLP reaction mixture (15 µL) comprised 3.5 μL of nuclease free water, 1 μL Fast digest buffer, 10 μL of the PCR product, and 0.5 μL of enzyme to make up a 15 μL total reaction volume. The digestion mixture was incubated at 37 °C for 30 min. Digestion products were resolved on 2% Ethidium bromide-stained agarose gels (Invitrogen, Carlsbad, CA, USA). Product sizes of 146 and 92 bp were expected for the wild type genotypes, and a 238 bp product for the variant type allele at the *C*-482 MspI restriction site. For the *T*-455 FokI restriction site, product sizes of 238, 134 and 104 bp for the wild type allele, four bands for a heterozygous allele and a 238 bp product for the variant allele were expected. Genotypes were assigned based on the pattern of restriction fragments and were classified as CC (homozygous wild), CT (heterozygous) and TT (homozygous) for the −482 restriction site; and TT (homozygous wild), TC (heterozygous) and CC (homozygous) for the *T*-455 restriction site.

Sequencing of a subset of the amplified products was done to corroborate the RFLP profiles. Twelve representative DNA fragments were selected according to restriction profiles and sequenced on both strands with primers used in the PCR reaction on a BigDye Terminator Cycle Sequencer. Nucleotide sequences were edited with the DNASTAR software (Seqman 8.1, Madison, WI, USA). Test sequences and wild type reference sequences were aligned with the BioEdit programme [38] for identification of nucleic acid variations.

### 4.4. HLA-B57 Gene Amplification, Sequencing, and Analysis

The PCR and cycling conditions for HLA-B57 amplification were as for *APOC3* amplification. However, the primers for HLA-B57 PCR were HLAF 5'-GAGTTTCACTTCTTCTCCCAAC-3' (sense) and HLAR 5'-GAAAATTCAGGCGCTTTGCA-3' (antisense). This gave a product of approximately 1729 bp. PCR products were purified and directly sequenced on both strands with primers: HLAF 5'-GAGTTTCACTTCTTCTCCCAAC-3' (sense), HLAR 5'-GAAAATTCAGGCGCTTTGCA-3' (antisense), HLAFb 5'-ACCCGGTTTCATTTTCAGTT-3' (sense) and HLARb 5'-TGGCCTCAACTGAAAATGAA-3' (antisense). Allele assignment was performed using the Assign Sequence Based Typing Software (Assign SBT 3.2.7, Conexio Genomics, Western Australia, Australia) and an HLA library compiled from the 2.17.1.0 release of the IMGT/HLA Database (www.ebi.ac.uk/ipd/imgt/hla/).

### 4.5. Statistical Analyses

Data was analyzed using the SAS statistical analysis package (SAS Version 8, SAS Institute Inc., Cary, NC, USA) and the student *t*-test. Descriptive statistics was used for all study participants. Analysis of variance (ANOVA) and Tukey’s test were used for multiple comparisons; Mauchly’s sphericity test was used for covariance determination (available in SAS statistical analysis package version 8). Allelic frequencies were estimated by the gene-counting method. The sample-size dependent standard error of alleles was calculated in terms of 95% confidence intervals of the estimates. Chi-square goodness-of-fit was used to verify the agreement of the observed genotype frequencies with those expected under the Hardy-Weinberg equilibrium [39]. A *p* value of <0.05 was considered statistically significant.

## 5. Conclusions

The relatively high prevalence of *APOC3* *T*-455CC genotype may suggest the importance of genetic testing prior to therapy initiation to pre-empt therapy associated lipid disorders. Overall, given the low frequencies of HLA-B*57:01 identified here and in other African populations, it may be important to screen patients at the individual level for abacavir sensitivity only when it is required, rather than at the population level. However, subsequent studies with a larger population size applying genome wide association analyses are needed to support these findings.
